# Rhythmic TMS Causes Local Entrainment of Natural Oscillatory Signatures

**DOI:** 10.1016/j.cub.2011.05.049

**Published:** 2011-07-26

**Authors:** Gregor Thut, Domenica Veniero, Vincenzo Romei, Carlo Miniussi, Philippe Schyns, Joachim Gross

**Affiliations:** 1 Centre for Cognitive Neuroimaging, Institute of Neuroscience and Psychology, University of Glasgow, Glasgow G12 8QB, UK; 2 Cognitive Neuroscience Section, IRCCS San Giovanni di Dio Fatebenefratelli, 25125 Brescia, Italy; 3 Department of Biomedical Sciences and Biotechnology, National Institute of Neuroscience, University of Brescia, 25123 Brescia, Italy; 4 Wellcome Trust Centre for Neuroimaging at UCL, Institute of Neurology, University College London, London WC1N 3BG, UK; 5 UCL Institute of Cognitive Neuroscience, University College London, London WC1N 3AR, UK

## Abstract

**Background:**

Neuronal elements underlying perception, cognition, and action exhibit distinct oscillatory phenomena, measured in humans by electro- or magnetoencephalography (EEG/MEG). So far, the correlative or causal nature of the link between brain oscillations and functions has remained elusive. A compelling demonstration of causality would primarily generate oscillatory signatures that are known to correlate with particular cognitive functions and then assess the behavioral consequences. Here, we provide the first direct evidence for causal entrainment of brain oscillations by transcranial magnetic stimulation (TMS) using concurrent EEG.

**Results:**

We used rhythmic TMS bursts to directly interact with an MEG-identified parietal α-oscillator, activated by attention and linked to perception. With TMS bursts tuned to its preferred α-frequency (α-TMS), we confirmed the three main predictions of entrainment of a natural oscillator: (1) that α-oscillations are induced during α-TMS (reproducing an oscillatory signature of the stimulated parietal cortex), (2) that there is progressive enhancement of this α-activity (synchronizing the targeted, α-generator to the α-TMS train), and (3) that this depends on the pre-TMS phase of the background α-rhythm (entrainment of natural, ongoing α-oscillations). Control conditions testing different TMS burst profiles and TMS-EEG in a phantom head confirmed specificity of α-boosting to the case of synchronization between TMS train and neural oscillator.

**Conclusions:**

The periodic electromagnetic force that is generated during rhythmic TMS can cause local entrainment of natural brain oscillations, emulating oscillatory signatures activated by cognitive tasks. This reveals a new mechanism of online TMS action on brain activity and can account for frequency-specific behavioral TMS effects at the level of biologically relevant rhythms.

## Highlights

► Rhythmic TMS bursts drive underlying brain rhythms ► This generates frequency- and site-specific patterns of oscillatory activity ► The enhanced rhythms reproduce natural brain oscillations ► A new mechanism of TMS action on brain activity and behavior has been revealed

## Introduction

As a method, transcranial magnetic stimulation (TMS) enables direct rhythmic stimulation of the human brain at frequencies that characterize electro- or magnetoencephalographic (EEG/MEG) signals [1, 2]. Likewise, the alternative method of transcranial alternating current stimulation (tACS) allows stimulation of the human brain at frequencies of biologically relevant brain rhythms [1, 2]. There is now accumulating experimental support that both rhythmic TMS and tACS interact with natural brain oscillations in a frequency-specific manner [3, 4, 5, 6, 7, 8]. This is based on findings that rhythmic stimulation of occipital or parietal areas results in specific (and immediate) perceptual consequences, when the stimulation frequency is tuned to the preferred oscillation frequency of the target area [3, 4, 5, 6, 7, 8]. (For analogous effects within the motor system, see [9].)

The above research provides new clues on two long-standing questions: (1) How does TMS (or tACS) interact with ongoing, here oscillatory brain activity to give rise to behavioral effects, and (2), what is the functional relevance of brain oscillations? It does so by pointing toward immediate and specific behavioral consequences depending on TMS (or tACS) frequency. However, these studies [3, 4, 5, 6, 7, 8] have one main limitation: they manipulated stimulation frequency (TMS or tACS) and reported behavioral outcome, but they did not study changes in brain activity, i.e., the underlying mechanisms.

Here, we present the missing piece to the puzzle of how these immediate, frequency-dependent effects on perception could come about during rhythmic TMS. Our study builds from the evidence that the behavioral effects of rhythmic TMS (or tACS) are confined to stimulation frequencies that were identified as perceptually relevant in prior EEG/MEG research [3, 4, 5, 6, 7, 8]. From this 1:1 frequency locking between the most effective TMS frequency and the perceptually relevant EEG/MEG frequency derives the hypothesis that rhythmic TMS pulses may have entrained the underlying rhythmic generator. This entrainment hypothesis therefore posits that frequency-tuned rhythmic TMS causes entrainment in direct interactions with the underlying brain oscillation. As a consequence, one of the mechanisms by which rhythmic TMS exerts its action on behavior could be the reproduction of a natural oscillatory signature of brain activity (that is also functionally relevant).

Entrainment supposes (1) the induction of a distinct entrainment signature, which emerges during rhythmic TMS and whose topography and frequency reproduce the natural oscillation of the targeted generator. Entrainment also supposes that there is (2) progressive enhancement of the target oscillation in the course of the TMS train as a result of progressive synchronization by each successive TMS pulse. Finally, entrainment should (3) depend on ongoing activity of the target generator, because it is supposedly driving existing brain oscillations, as opposed to generating new artificial rhythms.

We tested the entrainment hypothesis using neuronavigated rhythmic TMS of MEG-localized brain oscillators and concurrent multichannel EEG. We first identified a parietal α-oscillator (i.e., showing oscillatory activity at α-frequency, 8–14 Hz), whose EEG/MEG amplitude is regulated by visual attention [10, 11, 12, 13] and correlates with visual perception [13, 14, 15, 16]. We then tested in a passive condition whether rhythmic TMS of this parietal area at its preferred frequency entrains the underlying α-generator during TMS, explaining immediate and frequency-specific consequences of parietal α-TMS on performance in visual tasks [3, 4, 5]. Our results are in line with the three main predictions of the entrainment hypothesis. Rhythmic TMS therefore causes entrainment in direct interactions with the underlying brain oscillations.

## Results

For each participant, we first conducted a parietal α-source localizer experiment using MEG [10, 11, 12, 13]. On the basis of individual source estimates in structural magnetic resonance (MR) images and using TMS neuronavigation, we then targeted the most prominent (individual) α-source and tuned TMS to its preferred (individual) α-frequency. Accurate placement and 1:1 frequency locking served to create ideal conditions for entrainment (for modeled interaction between stimulus parameters and oscillators, see e.g. [17, 18]).

### Experimental Testing

We then ran four TMS conditions per participant while recording 62-channel EEG. In all conditions, we stimulated the same cortical α-generator with short TMS bursts (five pulses). In the main condition, we stimulated at individual α-frequency (α-TMS). During α-TMS, we oriented the TMS coil to induce currents perpendicular to the target gyrus, to maximize TMS efficacy (effect strength is enhanced with this coil orientation [19, 20, 21, 22]). In addition to α-TMS, we ran three control conditions. In one control (called arrhythmic TMS or ar-TMS), we applied the same number of TMS pulses as in α-TMS within the same time window (same mean frequency), but with randomly jittered interpulse intervals, holding all other TMS parameters constant. This control was meant to establish that the EEG signature to α-TMS does arise from rhythmic stimulation per se, and not from a basic response of α-generators to rapid-rate TMS bursts. In α-TMS_90_, we rotated the coil by 90° relative to α-TMS while holding the stimulation site constant (coil handle perpendicular versus parallel to the stimulated gyrus in α-TMS versus α-TMS_90_). Based on the dependence of TMS efficacy on coil alignment (and hence orienting of induced current) relative to the underlying gyral folding pattern [19, 20, 21, 22], we expected strongest entrainment for α-TMS relative to α-TMS_90_, whereas unspecific effects should be identical in the two conditions. Comparing α-TMS with α-TMS_90_ therefore allowed us to distinguish entrainment effects on neural tissue under the coil (the stimulated α-generator) from any unspecific effect that could be associated with rhythmic TMS (e.g., monitoring, TMS discomfort, rhythmic auditory clicks). Finally, we oriented the TMS coil in a sham position (α-TMS_sham_) to emulate the sound clicks associated with TMS without direct transcranial stimulation, to control for entrainment through rhythmic sounds. For further discussion on the choice of control conditions, see Note S1 in the Supplemental Discussion available online).

To rule out that EEG results could be of artifactual origin (note the use of a TMS-compatible EEG system [23] and artifact removal procedures; see Experimental Procedures), we also performed control TMS-EEG recordings in a phantom head using the same TMS, EEG, and postprocessing parameters as in the volunteers.

Below, we first describe localization of the parietal α-generator and how we placed the coil for the different conditions of TMS-EEG testing. We then describe the three key findings supporting TMS entrainment in the simultaneously recorded EEG: entrainment signature, progressive enhancement, and dependence on ongoing oscillations.

### Parietal Target Site: Identification of α-Generator and α-Frequency via MEG

The MEG α-localizer task involved attention orienting to the left or right in anticipation of a lateralized target. Behaviorally, this led to better performance at cued versus uncued positions (main effect of cueing validity: F_1,7_ = 12.53; p = 0.009), independent of target side (cueing validity × side: F_1,7_ = 2.99; p = 0.127). Participants responded significantly faster at attended (mean = 738.19 ms) than unattended locations (mean = 911.68 ms).

**Figure 1:**
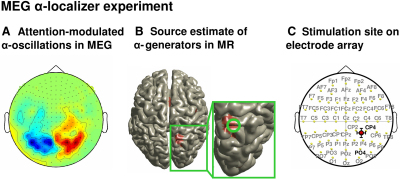
Identification of Parietal Target Site (A) MEG grand average to the localizer task in sensor space (covert rightward minus leftward orienting of attention). (B) Estimate of most prominent α-source leading to map in (A) (right hemisphere only). The source is projected on the standard MNI brain. (C) Average position of the TMS target site projected on the electrode array (international 10–20 EEG system) that was used for EEG recordings concurrently to five-pulse TMS bursts. The TMS hot spot (coil center) was located in between CP2, CP4, P2, and P4 (closest to CP4).

On the MEG sensor level, comparison between the two conditions (leftward versus rightward covert attention shifts) revealed in each participant the known α-signature of attention orienting (see Figure 1A for the grand average, comparable to e.g. [24]): a left-right mirror-imaged topography of α-power suppression (in blue) versus enhancement (in red) over parieto-occipital sensors when the two conditions are subtracted (attending rightward minus leftward). Anatomically, the underlying α-generators were localized in parietal areas of the attention network bilaterally (as revealed by maxima of t statistics in individual source space). TMS was then neuronavigated per participant to the right-sided, individual α-source. On average, this site was located at 31.3 ± 2.3, −63.5 ± 3.5, 60.3 ± 3.0 in Talairach space (x, y, z, ± standard error of the mean [SEM]) (see also Figures 1B and 1C). This location is near (1.5 cm Euclidian distance) a previous parietal target site (x = 17, y = −65, z = 54) measured in a functional magnetic resonance imaging (fMRI) study on attention orienting [25], and whose stimulation by α-TMS modulated perception [5]. Note that TMS of this site did not evoke phosphenes in any of our participants, which corroborates its location in higher-order attention areas (as opposed to low-level visual areas). The average individual α-frequency across all participants was 10.1 ± 0.31 Hz (±SEM) (range 9–11 Hz).

For α-TMS, we oriented the coil for each participant such that its handle pointed along the sagittal plane (upward), and currents were induced in anterior-posterior (y axis) and inferior-superior directions (z axis). In contrast, during α-TMS_90_, we oriented the coil such that its handle was pointing along the axial plane (sideways) and currents were induced in the left-right direction (x axis). These coil orientations were perpendicular (α-TMS) versus parallel to the target gyrus (α-TMS_90_), as indicated by Figure 1B (inset, see gyral folding pattern near the average α-source in the MNI brain) and were confirmed by analysis of individual MRI scans (see Note S2 in Supplemental Discussion).

### Entrainment Signature: Parietal α-TMS Bursts Mediate α-Power Enhancement at the Target Site

**Figure 2:**
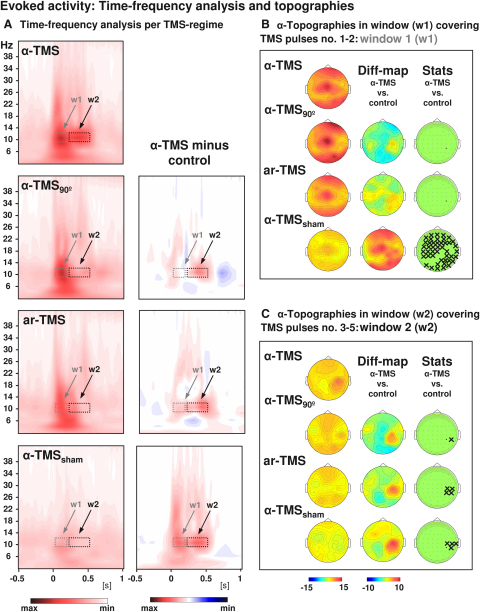
Grand-Averaged Time-Frequency Plots and Topographical Analysis Comparison of α-TMS bursts (active α-TMS perpendicular to target gyrus) with all three control conditions, i.e., α-TMS_90_ (active α-TMS parallel to target gyrus), ar-TMS (active rapid-rate TMS in an arrhythmic regime perpendicular to target gyrus), and α-TMS_sham_ (inactive α-TMS). (A) Time-frequency plots for electrode CP4 (closest to TMS hot spot) for all conditions (left panels) and subtractions (α-TMS minus control, right panels). w1 and w2 indicate windows of distinct early and late effects (the windows cover the entire train, which lasted 400 ms). (B) Topographies of the TMS-evoked responses for α-layer activity in the early window (w1). (C) Topographies of the TMS-evoked responses for α-layer activity in the late window (w2). The columns represent grand-average maps (left column), difference maps (α-TMS minus controls; middle columns), and corresponding t statistics (right columns). Xs indicate electrodes with statistically significant voltage differences in α-TMS relative to the corresponding control.

TMS entrainment supposes that frequency-tuned rhythmic TMS triggers a local oscillation at the target site, which cycles at the natural frequency of the targeted generator. In agreement with entrainment, time-frequency (TF) analysis of EEG during α-TMS (TMS-locked averages) shows prominent α-power boosting in a narrow α-band centered on the stimulation frequency, which was emerging over pulses 3–5 following an initial broadband response (shown for CP4 in Figure 2A, α-TMS) and was topographically restricted to the target site (Figure 2C, α-TMS). This narrow α-power boosting was absent in all three control conditions (Figures 2A and 2C, α-TMS_90_, ar-TMS, and α-TMS_sham_) and was not observed in recordings in a phantom head (see Note S3 in Supplemental Discussion and Supplemental Results). The latter result indicates that α-boosting during α-TMS was of neuronal origin, and not of artifactual nature.

At the beginning of the train, TMS bursts evoked brain activity over a large spectrum of frequency bands, including in a broad α-band (8–14 Hz) and in the θ- (∼4 Hz) and upper β-bands (∼25–30 Hz). These responses were transient, i.e., only present for the initial 1–2 pulses of the train (see Figure 2A, window 1 [w1]), and were condition unspecific because they were observed not only during α-TMS but also in the control conditions (Figure 2A, left panels; compare α-TMS with α-TMS_90_, ar-TMS, and α-TMS_sham_). In terms of topography (see Figure 2B, left scalp topographies representing α-layer activity), these early condition-unspecific responses were widespread, including frontocentral and bilateral occipital activity (see active TMS conditions: α-TMS, α-TMS_90_, and ar-TMS). Correspondence between conditions in window w1 is further illustrated in Figures 2A and 2B by only weak differences between α-TMS and control conditions in time-frequency representations (Figure 2A, right panels, difference plots) and in topography plots (Figure 2B, middle scalp topographies, difference maps [diff maps]). These differences were all insignificant (Figure 2B, right scalp topographies, stats) except, as expected, for the comparison with inactive TMS (α-TMS_sham_), where the differences reached significance for many electrodes (t statistics).

Critically, widespread broadband responses were absent in the second half of the train, where condition-specific responses emerged over pulses 3–5 in a narrow α-band and over the right parietal target site (Figures 2A and 2C, window 2 [w2]). These condition-specific responses were confined to α-TMS bursts, because no such α-power enhancement existed in the control conditions (α-TMS_90_, ar-TMS, and α-TMS_sham_). In addition, they were centered on a narrow α-band corresponding to the preferred oscillation of the targeted generator and hence the TMS frequency (average of ∼10 Hz; Figure 2A, upper left panel). Comparisons between conditions (subtraction and t statistics) confirmed strongest α-enhancement in w2 during α-TMS relative to all control conditions (Figure 2A, right panels), which localized over the right parietal target site (Figure 2C, middle scalp topographies for difference maps, right topographies for stats). Note in Figure 2A (upper panel) that the narrow α-band activity evoked by α-TMS is short-term, disappearing shortly after discontinuation of the train (∼100–150 ms later).

### Progressive Enhancement of α-Oscillations during α-TMS Bursts

**Figure 3:**
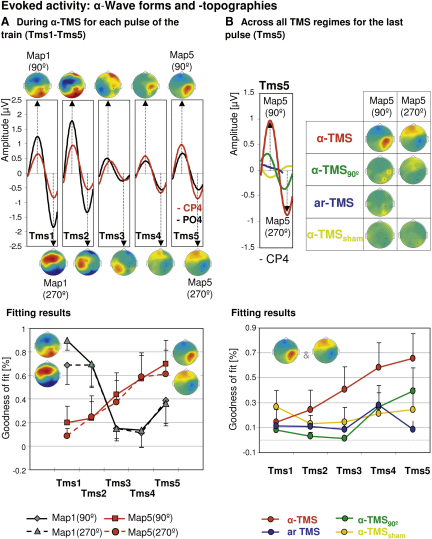
Evoked Activity: α-Wave Forms and Topographies (A) Top: α-waves and topographies in response to each successive single pulse during α-TMS. Waveforms are shown for electrodes CP4 (red) and PO4 (black). Map topographies are shown for the first and second part of the α-cycle (at 90° and 270° phase angle). Bottom: result of the spatial fitting procedure (spatial map correlations) for statistical evaluation of the time course of the initial (Map 1_90°&270°_) and end maps (Map 5_90°&270°_). (B) Top: α-waves and topographies to the last pulse of the train (Tms5) across all conditions. Bottom: result of the spatial fitting procedure (spatial map correlations) for statistical evaluation of the end α (entrainment) maps (Map 5_90°&270°_) across pulses (Tms1–5) per condition (α-TMS, α-TMS_90_, ar-TMS, and α-TMS_sham_). Error bars represent standard error of the mean.

Although the time-frequency analysis above reveals that α-TMS evokes a response in the α-band, it does not show whether this response is a true α-oscillation (i.e., a wave evolving over one full α-cycle or more). An alternative response could be an evoked component that simply repeats at α-rate but does not constitute a full α-wave. If TMS triggers α-oscillations, TMS-induced responses should show a cyclic pattern. With the addition of other phase-aligned TMS pulses (such as with α-TMS), this α-oscillation should then become progressively enhanced (synchronizing the activity of the underlying generator). To understand the nature of the evoked responses (α-waves versus components occurring at α-rate), we filtered the EEG signal to isolate the α-band and calculated an evoked α-response to each of the five successive TMS pulses of each condition. In agreement with entrainment, the results revealed that α-TMS (but none of the control conditions) triggered α-waves with a progressive time course over the right parietal target site (Figure 3).

Figure 3A (upper panel) represents α-responses to each single pulse of the main condition (α-TMS). The five waveform plots depict responses to each of the five successive TMS pulses Tms1–Tms5 (see boxes; electrodes CP4 [in red] and PO4 [in black] are superimposed). Each one of the five waveform plots reveals a clear cyclic pattern, with α-peak topographies at 90° α-phase angle (above waveforms) and 270° α-phase angle (below waveforms) showing inverted polarity but otherwise identical topography. Corroborating the time-frequency results of α-TMS above, topographies showed widespread α-responses to the initial 1–2 pulses (Tms1 and 2) (frontocentral and occipital maxima), followed over pulses 3–5 (Tms3–5) by more local α-responses close to the stimulation site (right parietal maxima) (Figure 3A, upper panel). For statistical comparisons, we then fitted the α-maps evoked by the initial (Tms1: Map 1_90°&270°_) and last TMS pulse (Tms5: Map 5_90°&270°_) to individual data using spatial map fitting procedures [26] (the fitting results are depicted in Figure 3A, lower panel). The results were analyzed via three-way analysis of variance (ANOVA) on goodness of fit (factors: map [Map 1 versus 5], consecutive TMS pulses [Tms1–5], and phase angle [90° versus 270°]). This statistically confirmed induction of α-oscillations and progressive enhancement at the target site. We found that α-maps at 90° versus 270° phase angle fitted the data equally well (no effect of phase angle or interactions; all p = nonsignificant), indicating cyclic activity. We found significant differences between initial and end maps (Map 1_90°&270°_ versus Map 5_90°&270°_) in terms of their time courses over the train [Figure 3A, fitting results; interaction map × TMS pulse: F(4,28) = 4.87, p = 0.004]. The occipitocentral α-maps (initial Map 1_90°&270°_) vanished after pulses 1–2 [Figure 3A, fitting results, gray lines, simple effect of TMS pulses: F(4,28) = 5.93, p = 0.0014]. In contrast, the right parietal α-maps (end Map 5_90°&270°_) progressively appeared over the successive pulses of the train [Figure 3A, fitting results, red lines, simple effect of TMS pulses: F(4,28) = 3.12, p = 0.03; polynomial linear contrast: 7.44, p = 0.029].

Comparing all conditions in terms of waves and topographies evoked by the end of the train (last pulse: Tms5, end Map 5_90°&270°_) (Figure 3B, upper panel) revealed that only α-TMS evoked a clear α-wave (left box, red line) and a right parietal α-map (right maps, compare α-TMS with α-TMS_90_, ar-TMS, and α-TMS_sham_; see Figure S2 for information across all pulses). Map fitting to individual data (fitting results depicted in Figure 3B, lower panel) statistically confirmed condition specificity. The right parietal (entrainment) maps (Map 5_90°&270°_ to α-TMS) showed a progressive time course during α-TMS, which was absent in all other conditions (Figure 3B, fitting results), as revealed by a significant interaction of condition × TMS pulses [F(12,84) = 2.02, p = 0.033] and follow-up polynomial linear contrasts. The latter were significant for α-TMS [F(1,7) = 7.44, p = 0.03], but not for any control condition [α-TMS_90_: F(1,7) = 2.97, p = 0.12; ar-TMS: F(1,7) = 0.43, p = 0.53; α-TMS_sham_: F(1,7) = 0.003, p = 0.95].

### Phase Alignment Depends on Ongoing Oscillations: α-TMS Bursts Phase Lock α-Oscillations as a Function of Pre-TMS α-Phase

TMS entrainment implies driving existing oscillatory processes of the brain. This should not only show in progressive synchronization of the stimulated generator to the TMS pulses (see above), but critically, this should depend on the momentary, ongoing oscillatory cycle of the underlying generator. This arises because TMS pulses will catch the stimulated generator at different stages of its cycle and therefore differentially amplify this endogenous oscillation. Specifically, entrainment performance should show a cosine-shaped function, with strong entrainment when TMS catches the ongoing oscillation at 0° and 360° of phase angle and weak entrainment at 180°.

**Figure 4:**
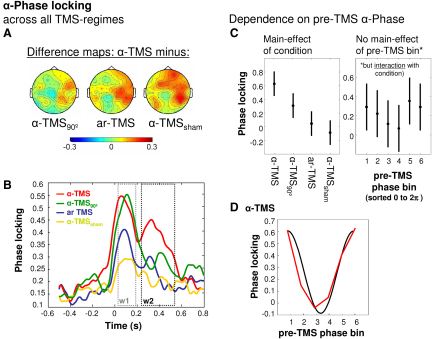
α-Phase Locking and Dependence on Pre-TMS Phase (A) Topographies of α-phase locking differences in window 2, expressed as change relative to baseline (left, α-TMS minus α-TMS_90_; middle, α-TMS minus ar-TMS; right, α-TMS minus α-TMS_sham_). (B) Time course of relative change of phase locking at significant electrodes with respect to baseline for all four conditions. (C) Results of two-way ANOVA on end α-phase locking (in window 2). Left: population marginal means for factor condition. Right: population marginal means for factor pre-TMS phase bin (sorted from 0 to 2π). Error bars represent 95% confidence intervals. (D) α-TMS-specific dependence of α-phase locking (w2) on pre-TMS phase bins. The black curve represents a perfect cosine function.

To fully detail the effect of our main condition (α-TMS) in terms of phase alignment and dependence on pre-TMS α-phase (and possibly α-amplitude), we computed the Hilbert transform of the band-pass-filtered (8–12 Hz) signal to obtain instantaneous phase and amplitude time series. To explore phase (Figures 4A–4D), we then calculated the phase-locking factor (PLF) [27]. PLF quantifies the consistency of the instantaneous phase across trials (with high values corresponding to high consistency). Figure 4A shows topographies of PLF differences in the α-band (α-PLF) during the second part of the TMS train (w2) between α-TMS and each control condition (left, α-TMS minus α-TMS_90_; middle, α-TMS minus ar-TMS; right, α-TMS minus α-TMS_sham_). Significant phase-locking increases in the vicinity of the stimulation site are evident (Figure 4A). The temporal evolution of α-PLF at significant electrodes (Figure 4B) shows an initial condition-unspecific increase in all conditions in window w1, followed by enhanced phase locking in window w2 during α-TMS (red line) as compared to the control conditions (green, blue, and yellow lines). This extends the results on evoked activity (Figure 2; Figure 3) by revealing that much of the condition-specific α-enhancement during α-TMS is due to increased phase locking in the course of the train. This is evidence for α-activity becoming more synchronized to the train, as opposed to unsynchronized activity increasing in power over the train.

Finally, we examined whether TMS-induced enhancement of PLF in w2 depends on pre-TMS α-phase (Figures 4C and 4D). We sorted pre-TMS α-phase (100 ms prior to TMS onset) into six equidistant bins. We computed mean α-PLFs for window w2 for each bin, condition, and participant and compared them in a two-way ANOVA. This revealed a significant main effect of condition [F(3,7) = 10.42, p = 0.001] and, importantly, a significant bin × condition interaction [F(5,3) = 1.85, p = 0.036]. The factor bin was not significant. Population marginal means illustrate the stronger phase locking during α-TMS compared to all other conditions (effect of condition, Figure 4C, left panel), but no main effect for bin (Figure 4C, right panel). Further analysis of the interaction revealed a significant dependence of PLF on pre-TMS phase only for α-TMS (high correlation with a cosine function, red versus black line, Figure 4D) (r = 0.92, p = 0.009 checked by bootstrap procedure).

Analysis of amplitude based on the Hilbert transforms reproduced the condition-specific differences in α-amplitude in the vicinity of the target site in w2 obtained above (α-TMS > all three control conditions; see Figure S3), but this α-enhancement in w2 did not depend on pre-TMS α-amplitude in a condition-specific way (no significant interaction between pre-TMS α-bin and condition; data not shown). Hence, condition-specific entrainment performance (during α-TMS) was dependent only on prestimulus α-phase, but not on prestimulus α-amplitude.

## Discussion

To understand the immediate effects of rhythmic TMS on perception, action, or behavior, it is necessary to identify the actions of TMS on brain activity. Here we reveal entrainment of brain oscillations in direct interaction with the underlying generator. We show local synchronization of parietal α-activity when targeting the underlying α-generator with TMS bursts at individual α-frequency (parietal entrainment signature). We demonstrate that this results from progressive synchronization of the underlying α-oscillator in the course of the TMS burst. Finally, we reveal enhanced synchronization (α-phase locking) to depend on the (pre-TMS) phase of the natural, ongoing activity of the stimulated generator. This suggests enhancement of a naturally occurring oscillation instead of the imposition of an artificial rhythm. In sum, our results show that short TMS bursts can drive natural brain oscillations by entrainment, one plausible mechanism via which TMS can act on the brain to modulate perception and performance.

Importantly, none of the three control conditions showed evidence of progressive α-synchronization, nor did recordings in a phantom head. First, there was no progressive α-synchronization with arrhythmic TMS bursts of the same duration and mean α-frequency (evidence against nonrhythmic α-generation by rapid-rate bursts). Second, there was no α-synchronization with sham TMS bursts that emulate the associated rhythmic auditory events (evidence against α-entrainment through auditory input, e.g., of multisensory neurons in parietal cortex). Third, there was no α-synchronization with rhythmic TMS bursts at suboptimal coil orientations inducing currents parallel to the gyrus (evidence against other unspecific TMS-effects, such as rhythmic somatosensory input). Fourth, it is important to emphasize that the stimulated α-generator was localized in a higher-order area of the attention network, whose stimulation by TMS did not evoke any visual light sensation (phosphene), to rule out potential visual entrainment through a TMS-induced, flickering visual percept (e.g., [28]). This therefore discounts explanations other than entrainment in direct interaction with underlying neurons.

Our EEG data reveal that short bursts of α-TMS can upregulate the targeted α-oscillations in higher-order parietal areas of the attention network, reminiscent of the α-amplitude regulation by voluntary attention orienting [10, 11], but without the need to engage the participant in an active task. Could this TMS-entrained α-signature that emulates the natural oscillations in origin and frequency also be of functional relevance? Previous EEG research revealed that the occipitoparietal α-band activity, which is amenable to attention control, also scales with visual perception [13, 14, 15, 16] and visual cortex excitability [29]. Specifically, occipitoparietal α-power shows an inverse relationship with contralateral visual performance [13, 14, 15, 16, 29]. Subsequent behavioral TMS research showed that parietal TMS bursts at this α-frequency (but not at control flanker frequencies) suppress visual perception, or visual representation in contralateral space [4, 5]. The present study therefore provides the missing link between a perceptually effective TMS protocol (parietal α-TMS) [4, 5] and functional EEG activity (parietal α-oscillations) [13, 14, 15, 16, 29]. The TMS-induced parietal α-signature that we observed is therefore likely also of functional relevance. Creating oscillatory brain signatures by TMS can open novel avenues to study not only oscillatory network interactions by means of concurrent EEG but also their functional role in perception and cognition, the latter by exploring behavioral consequences of entrainment.

As to a mechanistic account, our finding of progressive α-phase locking to TMS as a function of ongoing α-activity strongly supports phase resetting of ongoing oscillations, akin to the model of generation of sensory evoked potentials [30, 31, 32]. As a consequence of phase resetting, each TMS pulse should trigger waves that cycle at the frequency of the targeted area (approximating an oscillation kernel of entrainment). Because frequency-tuned TMS is phase aligned to these waves, they should then become progressively enhanced. Notably, TMS-aligned waves at the beginning of the train not only cycled at α-frequency but also involved oscillatory activity in the θ- and β-bands. α-oscillations only prevailed toward the end. One explanation is that the initial TMS pulses might have phase reset a multitude of parallel generators located within the same stimulated network but each cycling at a distinct frequency. This would be in keeping with evidence that single TMS pulses can probe into the natural rhythms of the stimulated area [33, 34, 35] and that parietal β- and θ-TMS leads to frequency-specific perceptual consequences, albeit ones distinct from the outcome of α-TMS [6]. Alternatively, parts of the initial responses may reflect an unspecific effect of TMS that is not necessarily of oscillatory nature (such as emotional or cognitive appraisal of TMS at the start of the bursts) rather than a genuine TMS impact on underlying neurons (see also [36]). This may be supported by our finding that the responses to the initial TMS pulses spread over several areas (including frontocentral sites). Importantly, however, over the course of the TMS train and in accord with TMS entrainment, only α-oscillations became progressively enhanced, whereas none of the other initial responses were promoted by further pulses. Importantly, also, stimulation in an arrhythmic mode did not lead to entrainment. In this condition, TMS pulses were randomly jittered around a mean α-frequency (about 10 Hz) to cover nonharmonic frequencies of 7–13 Hz (Table 1), which is likely to disrupt or even interrupt entrainment as a result of pulses being out of phase. TMS entrainment can therefore be construed as a progressive recruitment of neuronal elements cycling at the target frequency to phase align their activity to TMS. Note that the duration of entrainment that we report is in full agreement with findings that the oscillatory EEG response to one single pulse (the approximated oscillation kernel) lasts for only about one to two cycles [33, 35], the decay constant of the entrainment effect that we observe. Note also that there is no evidence for entrainment in the literature on long-term EEG aftereffects of clinical repetitive TMS protocols, given the lack of a consistent match between affected EEG and applied TMS frequency [37].

**Table 1:** TMS Conditions

	TMS Mode	TMS Regime	TMS Pulses	Train Duration	Frequency	IPI	Coil Handle Relative to Target Gyrus	Coil Surface Relative to Scalp
α-TMS	active	rhythmic	n = 5	4 × IAF cycle	IAF	fixed to 1000/IAF	perpendicular	tangential
α-TMS_90°_	active	rhythmic	n = 5	4 × IAF cycle	IAF	fixed to 1000/IAF	parallel	tangential
ar-TMS	active	arrhythmic	n = 5	4 × IAF cycle	NA	jittered by 0.7 × IPI, 0.8 × IPI, 1.2 × IPI, 1.3 × IPI	perpendicular	tangential
α-TMS_sham_	inactive	rhythmic	n = 5	4 × IAF cycle	IAF	fixed to 1000/IAF	NA	radial

The following abbreviations are used: IPI, interpulse interval; IAF, individual α-frequency; NA, not applicable. Note: in the formula for calculating individual jitter in ar-TMS, IPI refers to individual IPIs of the rhythmic regimes (= 1000/IAF). The presentation order of the four resulting, variable IPIs was randomized within each trial.

In light of our positive findings, it is of interest that previous attempts to entrain EEG oscillations to a rhythmic TMS train were unsuccessful [38]. It is unlikely that this discrepancy is simply explained by our selection of individual parameters for careful targeting of the generators. Although individual source estimation and 1:1 frequency matching between applied and preferred α-frequency is likely ideal for obtaining entrainment, parietal α-TMS leads to frequency-specific perceptual consequences also with fixed, not strictly individualized (10 Hz) frequency [4, 5]. Physiologically, this may be explained by trial-by-trial fluctuations around the average individual α-frequency. In addition, computational work shows that the tight relationship between effective stimulation frequency and preferred frequency loosens up as stimulus intensity increases, meaning that with increasing stimulus intensity, entrainment may occur from a progressively larger bandwidth, albeit one centered on the natural frequencies of the stimulated cortex (giving rise to the so-called Arnold tongues) (e.g., [17, 18]). This suggests that entrainment should also work when TMS frequency is slightly off the individual α-frequency. One explanation of previous null results of entrainment may be the use of overly conservative artifact-removal procedures (e.g., independent component analysis, as in [38]), as may be required in certain EEG devices with slow recovery times after TMS. This may eliminate not only the artifacts but also TMS-evoked activity. Alternatively, entrainment may have been complicated by the choice of a suboptimal coil orientation. Further research will need to study in detail the parameter space within which a natural oscillatory signature can be entrained.

### Conclusion

Our data show that short rhythmic TMS bursts can directly entrain underlying brain rhythms. This sheds new light on the direct interaction of rhythmic TMS with brain oscillations and significantly adds to an emerging literature on entrainment via alternative transcranial stimulation protocols, such as tACS [7, 8, 9, 39, 40, 41, 42]. Our data show that TMS entrainment can evoke spatially specific and frequency-specific signatures. The evoked signatures mimic naturally occurring, task-related modulations that are of functional significance. This may prove highly beneficial for the study of human brain oscillations.

## Experimental Procedures

### Participants

Ten healthy adult volunteers participated, of whom two had to be excluded from EEG analysis because of excessive eye blinks (artifacts) triggered by TMS. The remaining participants (five females, three males) had a mean age of 27.1 years (21–33 years) and were predominantly right handed by self report (one left handed). None had contraindications to TMS. All gave written informed consent prior to the study, which was approved by the ethics committee of the Faculty of Information & Mathematical Sciences of the University of Glasgow. The TMS protocol used accords with current safety guidelines and is of widespread use in studies on cognition [43].

### MEG Localizer Experiment

Participants performed in a symbolically cued visual-spatial attention-orienting paradigm, in anticipation of an upcoming, lateralized visual target. In short, a central visual cue of 0.2 s duration (randomly pointing either to the lower left or right visual field) prompted covert shifts of visual attention to the indicated position. After 1.7 s, the target appeared more often at cued than noncued locations (80% versus 20% of trials). The targets consisted of either an “x” or a “+,” whose luminance contrast with the background was chosen to give rise to perithreshold performance per participant. The participants were required to discriminate the two targets (giving left index responses for “x” and right index responses for “+”). There was a 3 s delay between the manual response and the next cue. Participants were asked to keep central fixation, to covertly direct and maintain attention to the cued position, and to respond to targets at cued and noncued locations.

MEG data of 100 trials were collected for each of the two attention conditions (randomly intermixed within five blocks of approximately 4 min duration each) using a 248-channel magnetometer whole-head MEG system (Magnes 3600 WH, 4-D Neuroimaging). Data were collected at 508 Hz sampling rate and online high-pass filtered at 0.1 Hz.

MEG data analysis focused on attention-related signals in the cue-target interval, normalized to baseline before cue onset. To this end, we epoched MEG signals time locked to cue onset (−1 to 2.5 s) and linearly detrended them prior to regression-based denoising using the signals from the reference sensors. Trials contaminated with artifacts were rejected after visual inspection. Spectral analysis was performed on two 1 s data segments (−1 s to 0 s and 0.5 s to 1.5 s) after applying Hanning tapers. Spectra for left-cue and right-cue trials were averaged separately and subtracted. The difference spectral plot was used to identify the individual α-generator (in the 5–15 Hz range) that showed strongest modulation by visual spatial attention.

Source localization was performed using dynamic imaging of coherent sources (DICS; [44]) on a 6 mm^3^ grid at individual α-frequency to localize the strongest generators of the α-modulation associated with the shift of spatial attention. To this end, a t statistic was computed for the single-trial difference between precue and postcue α-power. The 3D map of t values was visualized on the standardized structural MRI, and the coordinates of the global maximum were identified.

To control for adequate task performance, behavioral data were analyzed using repeated-measure ANOVA. Reaction times to targets were subjected to ANOVA with factors cueing validity (for targets appearing at cued versus noncued positions) and target side (left versus right). Post hoc tests were Bonferroni corrected. See Supplemental Experimental Procedures for more details.

### TMS-EEG

#### TMS Paradigm

TMS was applied at rest. Participants were comfortably seated with their chin positioned in a chin rest, their eyes open, and their gaze centered on a continuously displayed fixation cross (black on a gray background, RGB 192). Participants were asked to maintain central fixation and to minimize eye blinks and other movements during the recording blocks.

Short TMS bursts were delivered under the main (α-TMS) and three control conditions (α-TMS_90_, ar-TMS, and α-TMS_sham_). Each control condition differed in one aspect from the main condition. See text and Table 1 for details.

We neuronavigated the TMS coil (70 mm figure-of-eight coil connected to a Magstim Rapid2 Stimulator) in all conditions to the Talairach coordinates of the most prominent posterior α-generator of the right hemisphere (obtained from the MEG localizer task) via Brainsight (Rogue Research). Neuronavigation was based on individual source estimates and the individual structural MR scans.

TMS intensity was at 100% phosphene threshold, determined in blindfolded participants. Under blindfolding, occipital stimulation at experimental TMS intensity therefore evoked weak phosphenes in 50% of trials. With TMS over the α-generators that were localized in parietal cortex for all participants, no phosphene perceptions could be evoked (replicating [5]). The average stimulation intensity was 63.25% of maximum stimulator output (range 58%–66%).

The four TMS conditions were tested in a block design. In each block, 18 five-pulse TMS trains were given with an intertrain interval of 20 s, leading to 90 pulses per block over a block duration of about 6 min. All four conditions were tested in a series of four blocks (order of conditions randomized). This was repeated three times, leading to a total of 810 active TMS pulses with 54 trials per condition.

The duration of the experiment was 1.5–2 hr of recording, plus 1 hr for mounting the 64 EEG electrodes.

#### EEG Recording

Using TMS-compatible equipment (BrainAmp 64 MRplus, BrainProducts), EEG was continuously acquired from 62 channels (plus ground and reference electrodes). TMS-compatible sintered Ag/AgCl-pin electrodes were used. The signal was band-pass filtered at DC to 1000 Hz and digitized at a sampling rate of 5 kHz. Skin/electrode impedance was maintained below 5 kΩ.

#### EEG Analysis

Analysis was performed using the FieldTrip software package (http://fieldtrip.fcdonders.nl), custom-made MATLAB code, BrainVision Analyzer 1 (BrainProducts), and Cartool software (http://sites.google.com/site/fbmlab/cartool).

*Preprocessing and Artifact Removal.* Preprocessing epochs were of 4 s duration (−2 s to +2 s from TMS train onset). Epochs with excessively noisy EEG, eye movement artifacts (blinks or saccades), or muscle artifacts were excluded (mean acceptance rate 68%). The 50 Hz artifact was removed from remaining trials by fitting and subtracting a 50 Hz sine/cosine function. Subsequently, data were rereferenced to common average reference. After these steps, the remaining artifacts associated with the TMS pulses consisted of brief high-voltage peaks. These artifacts, which were generally of about 5–8 ms duration (replicating e.g. [23] using the same EEG equipment), were then removed using cubic interpolation for a conservative 15 ms interval following the TMS pulse. The same procedure was applied to remove the shorter and smaller rTMS recharge artifact [23], interpolating a 3 ms interval after ∼20 ms from each magnetic pulse (latencies varied across subjects according to TMS intensity). Single trials were carefully inspected to ensure absence of residual TMS artifacts. See also Note S3 in the Supplemental Discussion on TMS-induced artifacts.

*Wavelet Analysis across the Entire Epoch.* To analyze the oscillatory activity evoked by the TMS train, we calculated the average responses for an epoch of −0.5 s pre to +1 s post train onset and processed them with complex Morlet wavelets (2–40 Hz, 20 frequency steps, c = 5). Based on the results and in order to evaluate the entrainment of α-oscillations, we extracted the frequency range between 8 and 12 Hz for each subject from the wavelet dataset. Their spatial map topographies were plotted and compared across conditions (α-TMS versus controls) using subtraction plots (subtraction maps) and electrode-wise t statistics (t/p maps). The analysis revealed a biphasic response pattern (broadband response in an early time window, narrow α-band response in a later time window). Analyses were therefore performed on two windows (window w1 and w2), each centered on these responses. The early window (40–190 ms into the train) covered pulses 1–2, the later window (240–540 ms) pulses 3–5.

*Analysis of* α*-Waves to Each Successive TMS Pulse.* To analyze the spatiotemporal characteristics of α-waves, we band-pass filtered artifact-free data 8–12 Hz (Butterworth zero-phase filters, slope 48 dB/oct) and rearranged them to five epochs locked to the onset of each single pulse (epoch duration depending on individual α-frequency [IAF] and condition, minimum 0–90 ms post-TMS for IAF of 11 Hz, minimum 0.7 × 90 ms = 63 ms for ar-TMS). The average α-wave was then analyzed in terms of evolution of its topographies over the five pulses and in terms of differences across conditions. To this end, we performed spatial map fitting between the grand-average scalp topographies at the two α-peaks within an α-cycle (90° and 270°) and the corresponding individual α-peaks of the same or corresponding control conditions (intercorrelation between peak topographies). The fitting results were then analyzed to evaluate goodness of fit of grand-average maps (gmMap) in individual data to statistically secure their condition specificity (see e.g. [11] for application; see [26] for review). To this end, we computed ANOVAs on the fitting results (goodness of fit). To evaluate the evolution of α-waves across TMS pulses in the main condition (α-TMS), we conducted a 2 × 5 × 2 ANOVA with evoked map (start versus end), TMS pulse (Tms1–5) and phase (90° versus 270°) as within-subject factors. To compare α-waves across all conditions, we conducted a 4 × 5 × 2 ANOVA on fitting of map 5 with condition (α-TMS, α-TMS_90_, ar-TMS, and α-TMS_sham_), TMS pulse (Tms1–5) and phase (90° versus 270°) as within-subject factors.

*Analysis of Dependence on Ongoing Oscillations prior to TMS.* Artifact-free data were band-pass filtered 8–12 Hz and subjected to Hilbert transform for computation of instantaneous phase and amplitude. Intertrial phase locking was computed from the instantaneous phase ϕ as the absolute value of the mean of exp(i^∗^ϕ) across trials, also called phase-locking factor (PLF) [27]. PLF and amplitudes were individually averaged across trials (amplitudes were normalized by computing change relative to baseline [−500 ms to −100 ms]). Differences between conditions were tested for all electrodes in window w2. As a conservative significance estimate, we only considered electrodes with p < 0.05 (t tests) in all three comparisons (α-TMS minus α-TMS_90_, α-TMS minus ar-TMS, and α-TMS minus α-TMS_sham_) to be significant.

In a next step, trials were sorted into six bins of increasing pre-TMS α-phase (spanning from 0 to 2π) or pre-TMS α-amplitude (spanning from min to max) (100 ms before TMS onset). Ongoing α-activity prior to TMS showed a widespread distribution over occipital and parietal sites bilaterally (data not shown), in contrast to α-phase locking during TMS, which was spatially restricted to electrodes over the right parietal target site (Figure 4A), indicating local entrainment. The ongoing right parietal signal was therefore likely distorted by volume conductance from adjacent posterior sites (right occipital and left parietal). To decrease contributions from (possibly stronger) more distant α-sources prior to TMS and to thereby obtain a more reliable estimate of the ongoing α-phase and α-amplitude of the right parietal source (to be related to entrainment measures over right parietal sites), we computed pre-TMS phase and amplitude calculations for sorting on a bipolar montage (C4 − CP4) − (CP4 − P4). α-phase locking and α-amplitudes were averaged across window w2 and significant electrodes for each participant, condition, and bin separately before being subjected to 6 × 4 ANOVAs with factors bin and condition. Population marginal means of main factors were computed using the multcompare function in MATLAB (MathWorks).

To analyze whether the strength of phase locking was consistently modulated by pre-TMS phase, we computed the maximum cross-correlation between the six PLF values (one for each phase bin) and a cosine function for all four conditions (α-TMS, α-TMS_90_, ar-TMS, and α-TMS_sham_) and compared them to the 95th percentile of the null distribution for each condition separately. The null distribution was created by computing the maximum cross-correlation (across all lags) for 500 random permutations of the six PLF values. Significant cross-correlation was obtained only for α-TMS (Figure 4D).

## Supplemental Information

Document S1. Supplemental Results, Supplemental Discussion, Three Figures, and Supplemental Experimental Procedures
