# Supplementary material for: Rhythmic TMS Causes Local Entrainment of Natural Oscillatory Signatures
Source: Curr Biol. 2011 Jul 26;21(14):1176–85. doi: 10.1016/j.cub.2011.05.049 (PMC3176892; doi:10.1016/j.cub.2011.05.049)
Supplement: Document S1. Supplemental Results, Supplemental Discussion, Three Figures, and Supplemental Experimental Procedures [file mmc1.pdf]

Current Biology, Volume *21*

Supplemental Information

Rhythmic TMS Causes Local Entrainment of Natural Oscillatory Signatures

Gregor Thut, Domenica Veniero, Vincenzo Romei, Carlo Miniussi, Philippe Schyns, and Joachim Gross

Supplemental Discussion

Note S1: Choice of Control Conditions

The TMS entrainment hypothesis leads to clear predictions as to the associated EEG changes. The hypothesis predicts the emergence of an entrainment map, characterized by enhanced brain oscillations at the stimulation-frequency (frequency-specificity of entrained oscillation) and stimulation-site (location-specificity of entrained oscillation), when TMS bursts are tuned in frequency to the underlying oscillation. Three control conditions and recordings in a phantom head were implemented to test that this entrainment signature does not emerge as a consequence of other effects associated with TMS bursting (including artifacts).

We did not run additional conditions in which we stimulated the parietal cortex with rhythmic TMS at a non-α-frequency (other-frequency condition), or in which we stimulated adjacent sites with α-TMS (other-site condition), because these conditions are not instrumental for testing the entrainment hypothesis. This is because parietal neurons can oscillate in many different frequency bands, and α-oscillations occur across many areas of the brain (which could be isolated by other localizer tasks). By extension, there are no predictions as to the outcome of rhythmic TMS under these two experimental manipulations (other frequency and site). Studying these outcomes is of interest to learn more about brain oscillations, but not for testing the entrainment hypothesis.

Note S2: Individual Sulcal Patterns at the Individual α-Source

The location of the average α-source in the standard MNI brain (see main text, Figure 1B, inset for nearby gyral folding pattern) indicates that an upward coil orientation (handle) as in α-TMS is perpendicular to the target gyrus, whereas a sideways orientation as in α-TMS_{90} is parallel to it. For verification, we fitted in each individual scan a vector of norm 1 to the sulcus next to the individual α-source. On average, this sulcus was oriented sideways ($x=0.91\pm0.07$, $y=0.31\pm0.22$, $z=-0.08\pm0.16$; mean±SEM), with x significantly bigger than y and z ($p<0.001$ corrected t-tests). This confirms that the coil was consistently oriented perpendicular to the gyrus during α-TMS, and parallel during α-TMS_{90} also across individual subjects.

Note S3: TMS-Induced Artifacts

TMS-EEG is inevitably associated with TMS-induced artifacts in the EEG recording (Ilmoniemi and Kicic, 2010; Miniussi and Thut, 2010). We here used a TMS-compatible EEG system and settings (recording parameters, EEG electrodes, TMS devices) for which artifacts have been shown to be of short duration, i.e. about 6ms (Bonato et al., 2006, Veniero et al., 2009, Ferreri et al., 2010). This is replicated in our recordings showing artifact durations of 5-8ms. Hence, the device used brings artifacts down to 5-8ms post-TMS, which allows the tracking and interpretation of brain signals online to the TMS bursts, after artifact removal (see main text, methods for artifact removal procedure).

This is illustrated in one single subject with continuous raw data from the main (α-TMS) condition recorded over one electrode close to the TMS coil (CP4) (see Figure S1A). The figure illustrates unprocessed (raw) data (blue line), and data after artifact removal (green line). The main artifacts at TMS delivery are readily visible (5 vertical blue lines). Note the second, smaller TMS recharge artifact after the main artifact (smaller blue spike), occurring in this case at a delay of about 19ms from the TMS pulse (see also box inset in Figure S1A). Both these artifacts are eliminated by artifact removal, without affecting the EEG signal in between the brief, spike-shaped artifacts (see e.g. box inset, green line, Figure S1A). In the represented single-trial data, these signals consist of an induced α-oscillation, which emerges in the course of the 5-pulse TMS trains.

Could this α-band signal in the α-TMS condition represent a TMS-induced artifact? Several arguments speak against an artifactual origin (see no. 1-5 below), or in favor of a brain signal (no. 6): <u>First</u>, artifacts are not expected to progressively increase over time and to depend on on-going oscillatory activity. <u>Second</u>, electromagnetic artifacts are present to a similar degree in all active conditions (α-TMS, α-TMS_{90}, ar-TMS), i.e. are not expected to differ so substantially between conditions. <u>Third</u>, the TMS-induced artifacts in the employed TMS-device consist of brief, high-voltage and high-frequency peaks (around 200Hz) (as identified e.g. in previous phantom head recordings, Veniero et al., 2009), which is well outside the observed spectral peak in the α-frequency band (i.e. >> 10Hz). <u>Fourth</u> and by extension, these artifacts cannot account for the spectral peaks at a narrow α-band, centered on the TMS-frequency. <u>Fifth</u>, our artifact removal procedure does not at any stage involve filtering (only 50Hz-fitting and subtraction), and can therefore not induce filter-artifacts from some spike-shaped components, that might not have been completely eliminated.

Still, despite these arguments, the signal in the α-band might stem from an as yet unknown artifactual source that is linked to our artifact removal procedure, to α-TMS per se or an interaction of both (removal procedure and α-TMS). This could result from accidental transformation of a residual spike-shaped artifact into an α-component, or a resonant response in EEG-electrodes and wires induced by α-TMS. To rule this out, we recorded α-TMS-EEG in a phantom head (see Supplemental Results below), using the same EEG-, TMS- and analysis parameters as in our main experiment. The data did not reveal any observable α-band signal, hence demonstrating that the recorded α-band signal stems from the brain (<u>point no 6</u>), and is not of artifactual origin.

Supplemental Results

Control Experiment in a Phantom Head: Design and Data

To rule out a possible artifactual source of the induced α-oscillation, we recorded EEG in a phantom head (melon, Taxon: *Cucumis melo var. cantalupensis*, in analogy to Veniero et al., 2009). We mounted nine electrodes (C2, C4, C6, CP2, Cp4, CP6, P2, P4, P6) with the central electrode of this configuration (CP4) being stimulated by TMS. EEG settings were identical to those used with our participants (see main text, experimental procedures). Likewise, TMS was applied at comparable intensity (65% maximum TMS output) with the same coil orientation as in α-TMS (handle pointing upwards). To cover the range of individual α-frequencies tested in our participants, we ran three α-TMS conditions (9Hz, 10Hz, and 11Hz).

Contrasting single-subject and phantom data over an electrode under the TMS coil (CP4) (Figure S1) clearly shows that α-oscillations are induced only in the participants, but not the phantom head.

In single-subject data, TMS triggered oscillations are apparent both on the level of single trials (Figure S1A) and the cross-trial averages (Figure S1B) and prominently peak in the α-band (Figure S1C). In the represented single-subject data, induced α-activity peaked at 11Hz (see spectral plot in Figure S1C), which is also this participant's individual α- and TMS-frequency. Note also that the α-peak at pulse no. 5 (last pulse) was not only spectrally restricted to the α-band, but also topographically restricted to the parietal target site (Figure S1B, map inset).

In the phantom head, in contrast, no oscillation is apparent for α-TMS at any of the tested frequencies (9-11Hz), neither on the single-trial level (Figure S1D), nor at the level of cross-trial averages (Figure S1E). The spectral plots show a flat line, when scaled to the single-subject data (Figure S1F). Logarithmic scaling for comparison between single-subject and phantom data (see grey insets in Figure S1C vs. S1F) shows that oscillatory α-activity in the participant exceeds phantom data by a factor of 10'000 (as inferred from the power spectrum), and that no α-peak is visible above noise-levels in the phantom data. Hence, we conclude that the measured α-oscillation is of neuronal origin.

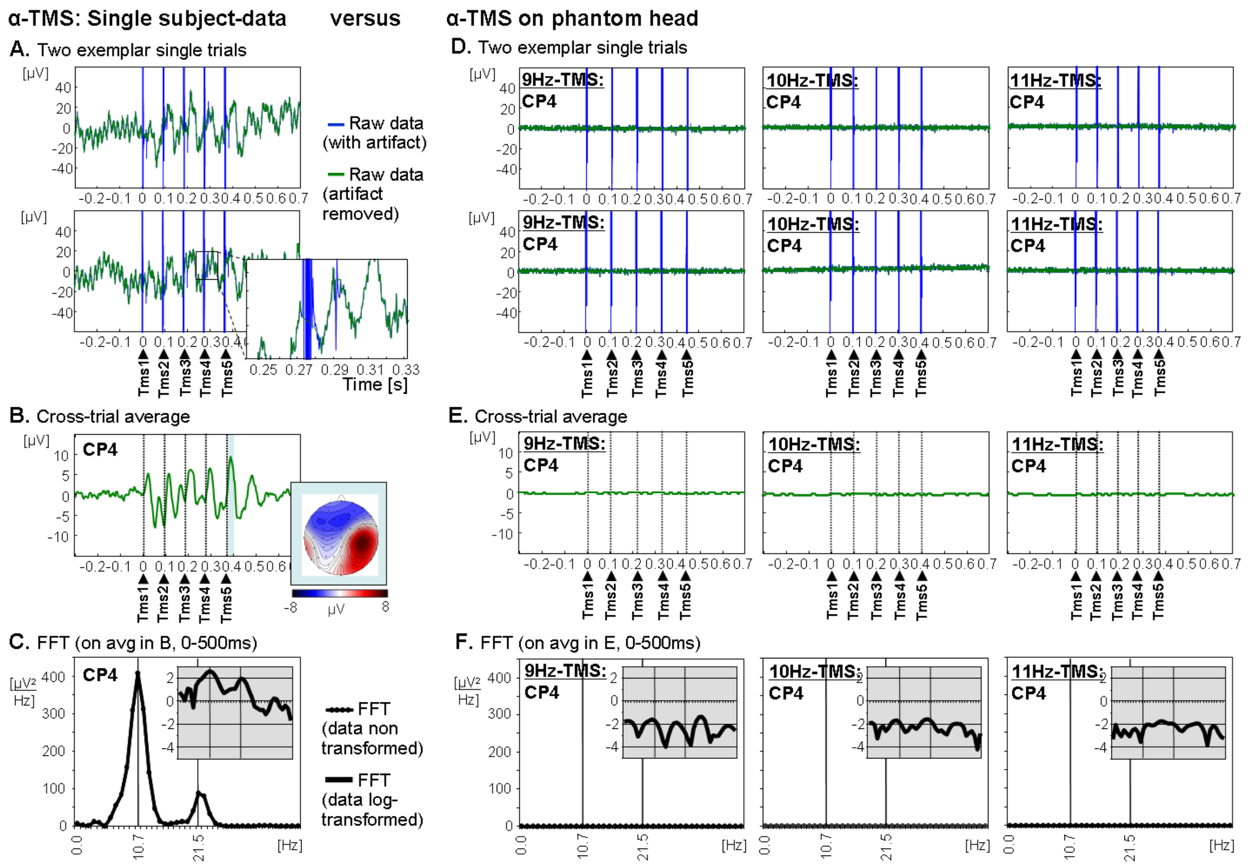

Figure S1. Single-Subject versus Phantom EEG Data for α-TMS

Raw data of 2 trials (A vs. D), cross-trial averages (B vs. E) and power spectrum of the averages (C vs. F).

(A, B, D, and E) An epoch of 1 sec of unprocessed data is shown before (blue line) and after artifact removal (green line), starting from 0.3sec before to 0.7sec after α-TMS burst onset. Short spike-shaped amplitude peaks (blue line) that coincide with the TMS pulses are visible in both single-subject and phantom data. These represent the main artifacts (that are eliminated by artifact removal procedures). To this adds TMS-triggered oscillatory activity that is only observed in the single-subject data (not the phantom) (green line).

(C and F) Power spectra show that this TMS-induced oscillation peaks at 11Hz, the individual α-frequency of the participant. Insets in C,F show log-transformed power spectra.

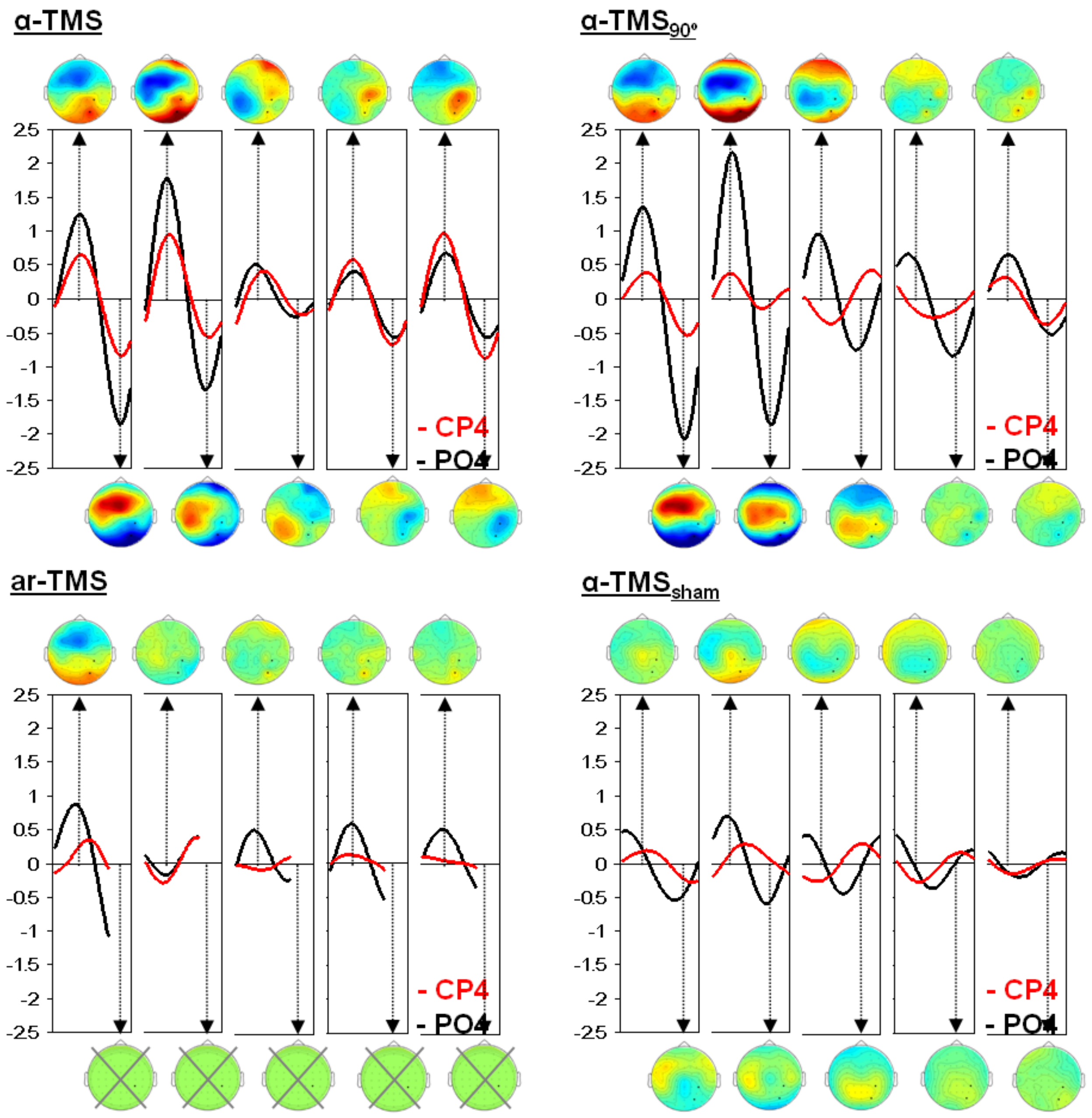

Figure S2. Evoked α-Waves (Forms and Topographies) across All Pulses (Tms1-Tms5) and Conditions, Related to Figure 3

Waveforms are shown for electrodes CP4 (red) and PO4 (black). Map topographies are shown for the first and second part of the α-cycle (at 90° and 270° phase-angle).

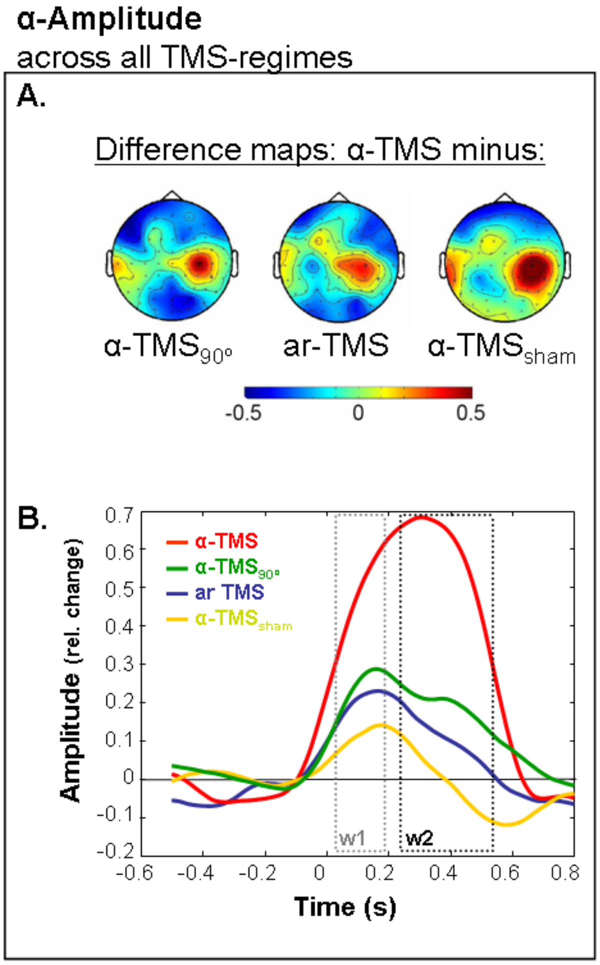

Figure S3. Amplitude Effects from Hilbert-Transformed Data, Related to Figure 4

(A) Topographies of α-amplitude differences in window 2 (left: α-TMS minus α-TMS_{90}, middle: α-TMS minus ar-TMS, right: α-TMS minus α-TMS_{sham}).

(B) Time course of α-amplitude at significant electrodes for all four conditions.

Supplemental Experimental Procedures

MEG Paradigm
Participants performed in a symbolically cued visual-spatial attention-orienting paradigm, in anticipation of an upcoming, lateralized visual target in the left or right visual field.

They viewed a central fixation cross (black, size: 0.8x1.2° of visual angle) and two placeholders (unfilled black squares, size: 3.2x3.2°) positioned in the lower left and right visual field (square centres were 8° below and 10° to the left or right from the fixation cross) on a grey background (rgb: 192). The placeholders marked the to-be-attended positions and target locations. Fixation cross and placeholders were presented continuously throughout the task, and the lines of the fixation cross were arranged to point in the direction of each square centre.

Each trial started with a spatial cue (one line of the cross changing to a dotted format for the duration of 200ms), randomly cueing either to the lower left or right placeholder. After 1700 ms, the target appeared, more often at cued than non-cued locations (80 vs. 20% of all trials). The targets (presented for 80ms in the centre of the placeholders) consisted of either an "x" or "+" (sizes: 0.6x0.6°) (1:1 probability of appearance). Target luminance was chosen to give rise to peri-threshold performance per participant (targets chosen from a set of stimuli with rgb values of 72, 96, 120, 144, 156, 168, with most subjects being tested at 168). Left index responses were given for "x" and right index responses for "+". There was a 3000 ms delay between the participant's response to the target and the next cue.

Participants were asked to keep central fixation, to covertly direct and maintain attention to the cued position, to respond to targets at cued and non-cued locations and to minimize eye blinks.

Each subject practiced the task prior to MEG-recording in a psychophysics lab booth outside the MEG (also allowing to control for correct task performance without overt eye movements), plus for one block inside the MEG at the start of the experiment. During the experimental session, 100 trials were presented for each of the two cue conditions (randomly intermixed within 5 blocks of approximately 4 min duration each). Target contrast was adapted in between blocks when required (as a consequence of ceiling or floor effects on perception). Visual stimuli were presented on a CRT monitor for training in the lab, and on a screen inside the shielded MEG room through a DLP projector (PT-D7700E-K, Panasonic). Behavioural data were collected via a response box (E-Prime; Psychology Software Tools, Pittsburgh, PA, USA) outside; and a non-magnetic response pad (Lumitouch) inside the MEG room.

MEG Recordings
A 248-magnetometer whole-head MEG-system was used (MAGNES® 3600 WH, 4-D Neuroimaging). MEG-signals were acquired in a magnetically shielded room at 508Hz sampling rate and online high-pass filtered at 0.1Hz. Before the recording session, five coils were attached to the participants' heads and their 3D-locations with respect to three anatomical landmarks (nasion, LPA, RPA) were recorded using a Polhemus system. Short activation of these coils at the beginning and end of each run allowed computation of the individual head position with respect to the sensor array. During the recording session, subjects were seated in a reclining chair, with their head supported against the back and top of the magnetometer. Participants were asked to remain as still as possible during the recordings and were continuously monitored by a video camera.

MEG Data Analysis

Analysis was performed using the FieldTrip software package (http://www.ru.nl/fcdonders/fieldtrip/) and custom-made Matlab code.

Preprocessing and Frequency Analysis

MEG-signals were epoched time-locked to cue-onset (-1 to 2.5s) and linearly detrended prior to regression-based denoising using the signals from the reference sensors. Trials contaminated with artifacts were rejected after visual inspection. Signals were transformed to planar gradient representation (horizontal and vertical component) and subjected to spectral analysis. Spectral analysis was performed on two 1s-long data segments (-1s to 0s and 0.5s to 1.5s) after applying hanning tapers. Spectra for left-cue and right-cue trials were averaged separately and subtracted. This difference spectral plot was used to identify the individual α-generator (in the 5-15Hz range) that showed strongest modulation by visual spatial attention.

Source Localization

Individual structural MRIs (obtained with a Siemens TrimTrio scanner) were normalized using a linear transformation in SPM2 (www.fil.ion.ucl.ac.uk/spm/). Subsequently, source localisation was performed on a standardized grid (6mm^3 voxel size) that was transformed into the individual head coordinate system. A beamforming method (DICS, Gross et al., 2001) was employed based on the cross-spectral density matrix at individual α-frequency to localise the strongest generators of the α-modulation associated with the shift of spatial attention. To this end, a *t*-statistic was computed for the single trial difference between pre-cue and post-cue α-power. The 3D-map of *t*-values was overlaid on the standardized structural MRI and the coordinates of the global maximum (in Talairach space) were identified, and then used for accurately positioning the TMS coil with the help of the neuro-navigation system.

Supplemental References

Bonato, C., Miniussi, C., and Rossini, P. M. (2006). Transcranial magnetic stimulation and cortical evoked potentials: a TMS/EEG co-registration study. Clin Neurophysiol *117*, 1699-1707.

Ferreri, F., Pasqualetti, P., Maatta, S., Ponzo, D., Ferrarelli, F., Tononi, G., Mervaala, E., Miniussi, C., and Rossini, P. M. (2010). Human brain connectivity during single and paired pulse transcranial magnetic stimulation. Neuroimage *54,* 90-102.

Ilmoniemi, R. J., and Kicic, D. (2010). Methodology for combined TMS and EEG. Brain Topogr *22*, 233-248.

Miniussi, C., and Thut, G. (2010). Combining TMS and EEG offers new prospects in cognitive neuroscience. Brain Topogr *22*, 249-256.

Oostenveld, R., Fries, P., Maris, E., Schoffelen, JM (2011) FieldTrip: Open Source Software for Advanced Analysis of MEG, EEG, and Invasive Electrophysiological Data. Computational Intelligence and Neuroscience, Volume 2011, doi:10.1155/2011/156869

Veniero, D., Bortoletto, M., and Miniussi, C. (2009). TMS-EEG co-registration: on TMS-induced artifact. Clin. Neurophysiol. *120*, 1392-1399.
